# Supplementary figures and images for: Mitochondrial redox adaptations enable alternative aspartate synthesis in SDH-deficient cells
Source: eLife. 2023 Mar 8;12:e78654. doi: 10.7554/eLife.78654 (PMC10027318; doi:10.7554/eLife.78654)

**Figure 1F**

WT and SDHB UOK269

Raw Image

WT UOK269

SDHB UOK269


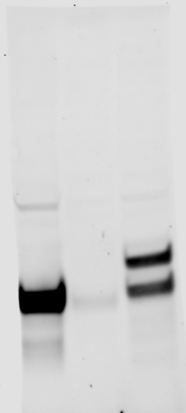

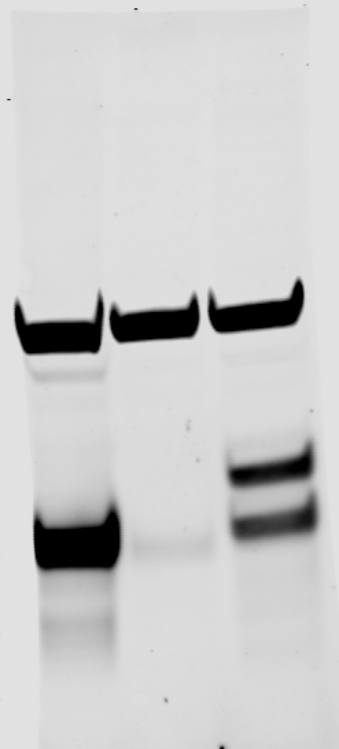

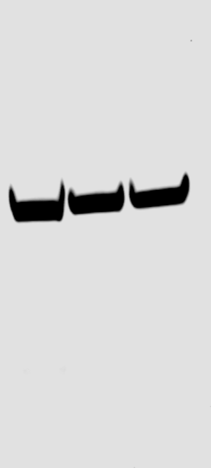


30 kDa

SDHB + V5

800 channel

680 channel

Tubulin

50 kDa

Supplement: Figure 1—source data 2. [file elife-78654-fig1-data2.zip › Figure 1-source data 2.docx]

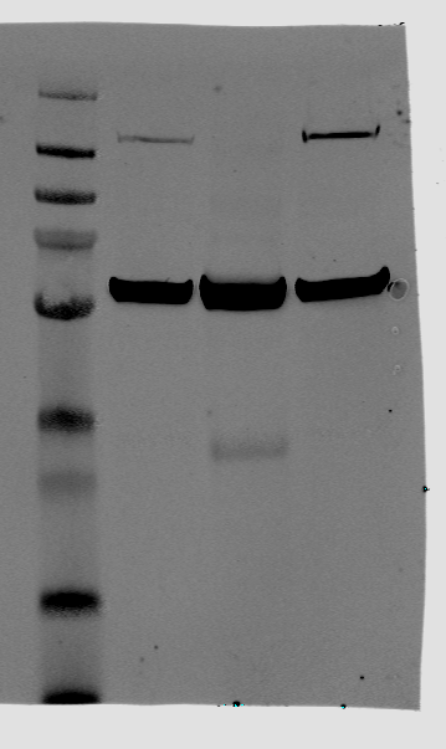


WT PC KO 2 PC AB 2

Tubulin

PC

**Figure 4G**

PC KO blot (WT 143B, PC KO 2, PC AB 2)

115 kDa

50 kDa

Raw Image

Supplement: Figure 4—source data 2. [file elife-78654-fig4-data2.zip › Figure 4-source data 2.docx]

**Figure 5A**

WT 143B, MPC1 KO clones 1-2, MPC1 AB 1


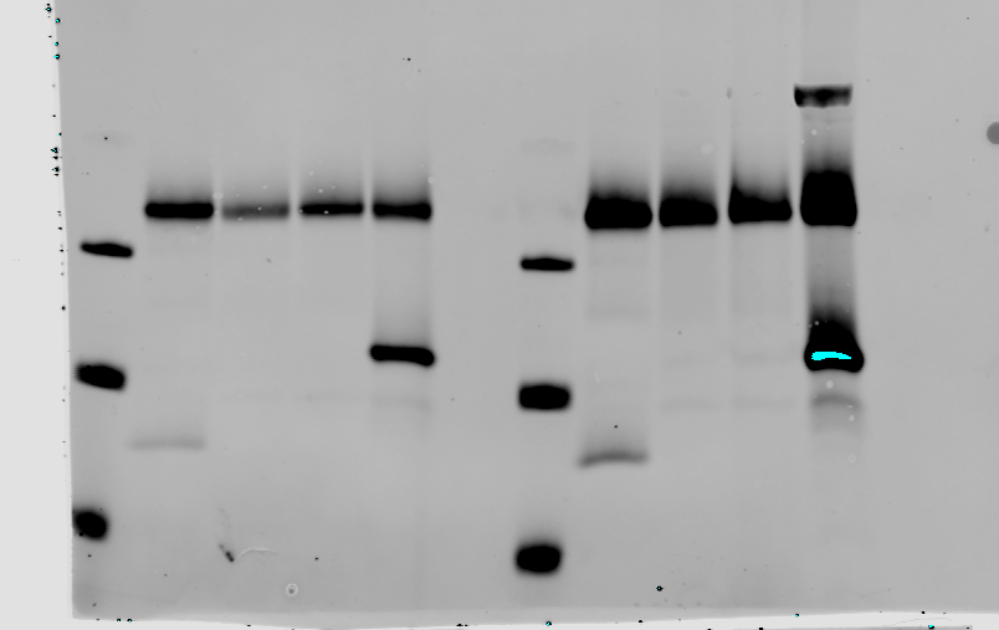


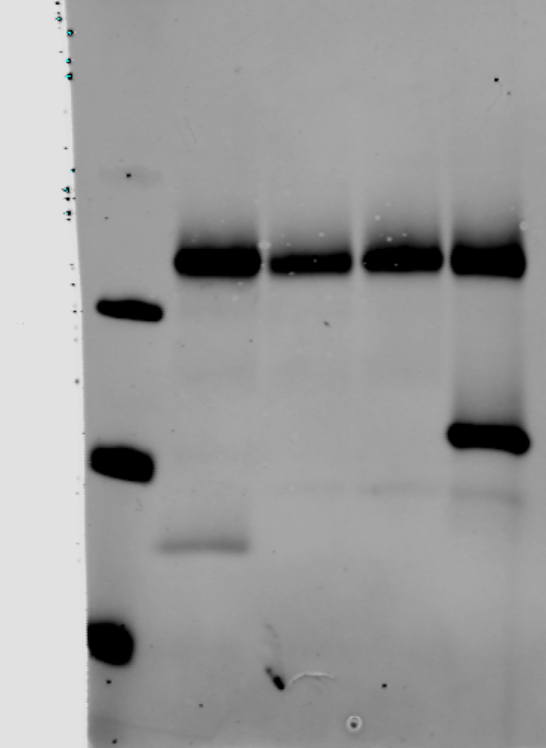
Raw Image

MPC1

WT KO 1 KO 2 AB 1

SDHB

MPC1-V5

25 kDa

15 kDa

10 kDa

Supplement: Figure 5—source data 2. [file elife-78654-fig5-data2.zip › Figure 5-source data 2.docx]

**Figure S6D**

MDH1 KO 143B blot


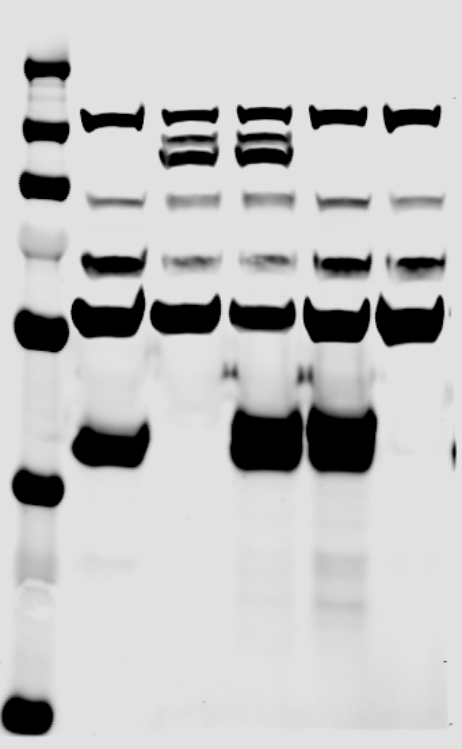


Raw Image


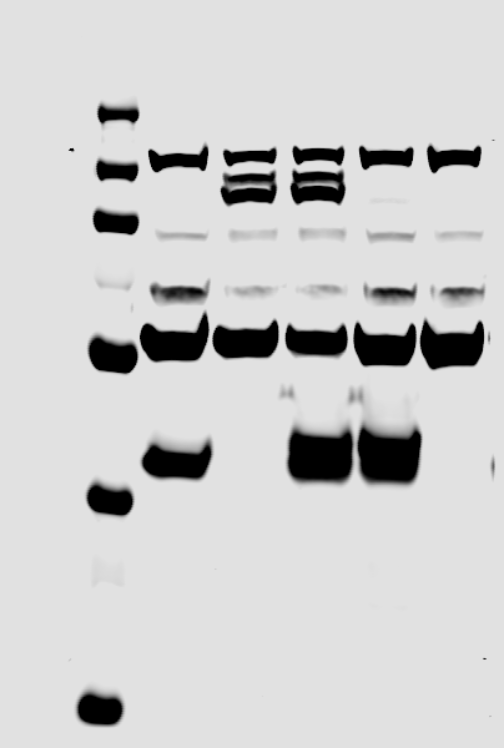


Vinculin

Tubulin

MDH1

MDH1 AB

MDH1 KO

WT

115 kDa

50 kDa

30 kDa

Supplement: Figure 6—figure supplement 1—source data 2. [file elife-78654-fig6-figsupp1-data2.zip › Figure 6-figure supplement 1-source data 2.docx]

**Figure 7I 143B** (NDUFA8)


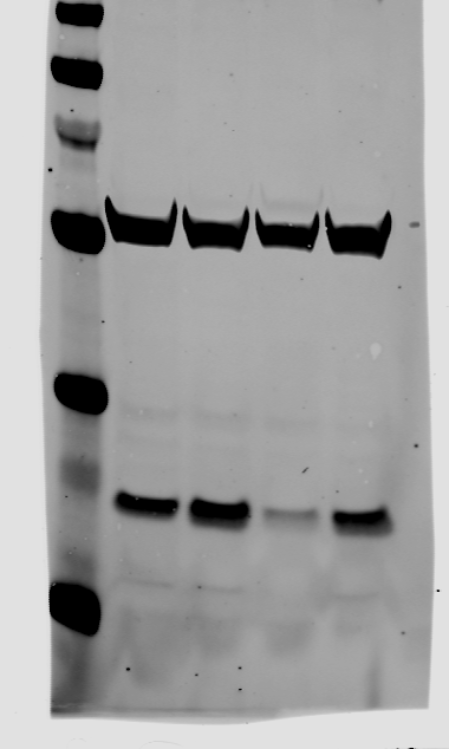


Raw Image


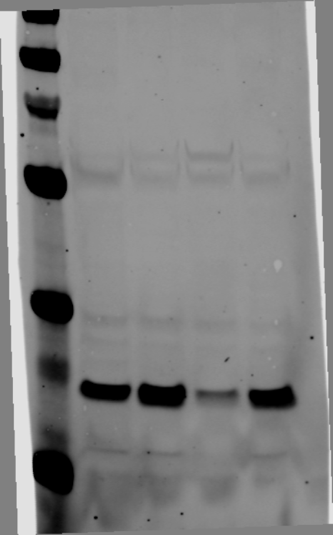

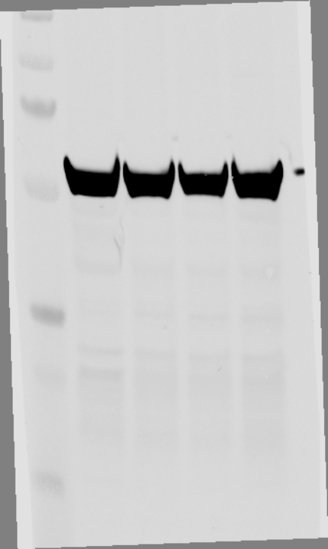


WT AB EP LP

NDUFA8

Tubulin

15 kDa

25 kDa

50 kDa

Supplement: Figure 7—source data 2. [file elife-78654-fig7-data2.zip › Figure 7-source data 2.docx]

**S7A**

WT 293T and SDHB KO 293T


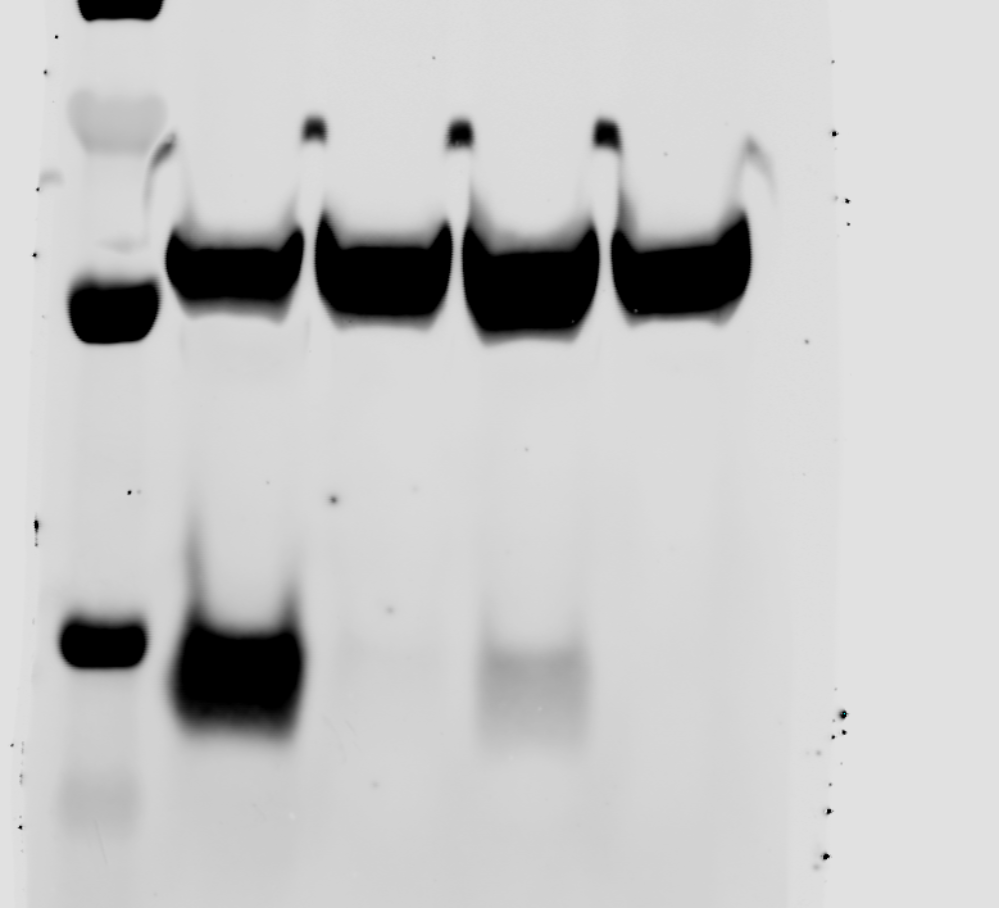


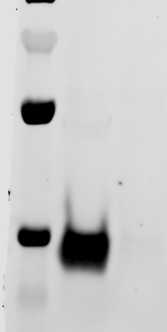

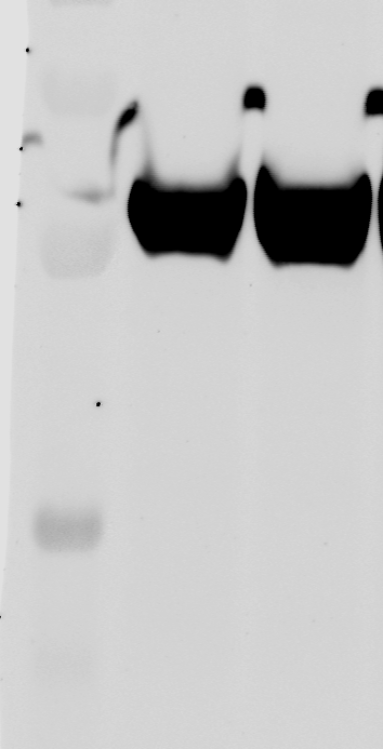


WT KO

Tubulin

SDHB

30 kDa

50 kDa

Supplement: Figure 7—figure supplement 1—source data 2. [file elife-78654-fig7-figsupp1-data2.zip › Figure 7-figure supplement 1-source data 2.docx]

**Figure 8A**

WT 143B, UOK269 and SDHB UOK269 (NDUFA8)


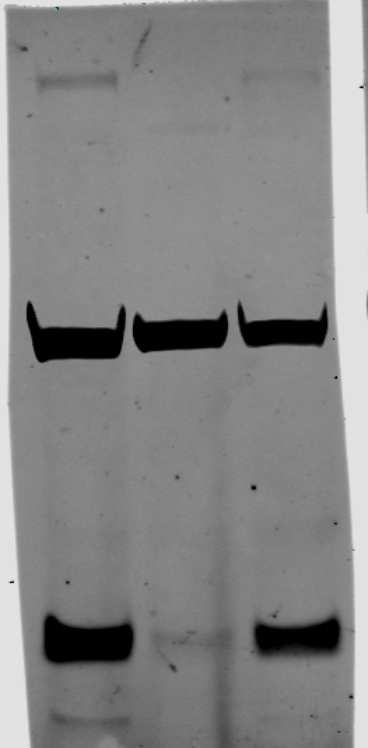


Raw Image


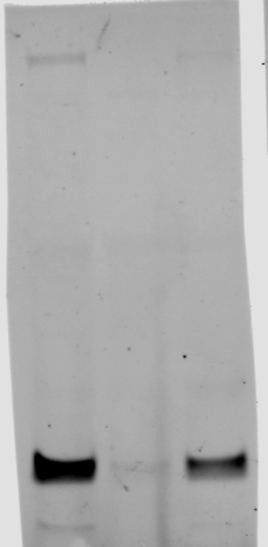

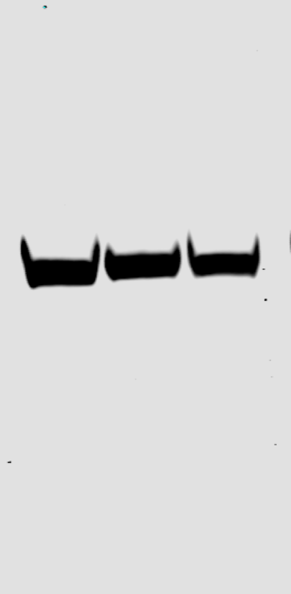


SDHB UOK269

UOK269

WT 143B

Tubulin

NDUFA8

25 kDa

50 kDa

Supplement: Figure 8—source data 2. [file elife-78654-fig8-data2.zip › Figure 8-source data 2.docx]
