# Supplementary material for: Mitochondrial redox adaptations enable alternative aspartate synthesis in SDH-deficient cells
Source: eLife. 2023 Mar 8;12:e78654. doi: 10.7554/eLife.78654 (PMC10027318; doi:10.7554/eLife.78654)
Supplement: Figure 1—figure supplement 1—source data 2. [file elife-78654-fig1-figsupp1-data2.zip › Figure 1-figure supplement 1-source data 2.docx]

Figure 1-figure supplement 1

**Figure supplement 1B**

WT 143B and SLC1A3

WT SLC1A3


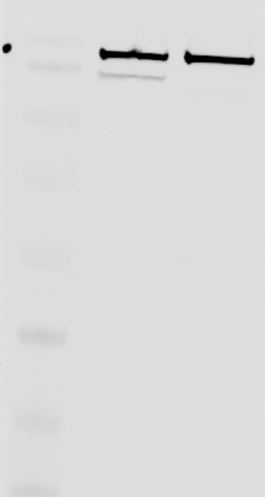

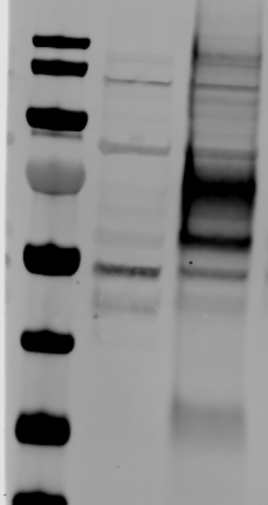


Vinculin

115 kDa

115 kDa

SLC1A3

60 kDa

680 channel

800 channel

Raw Image


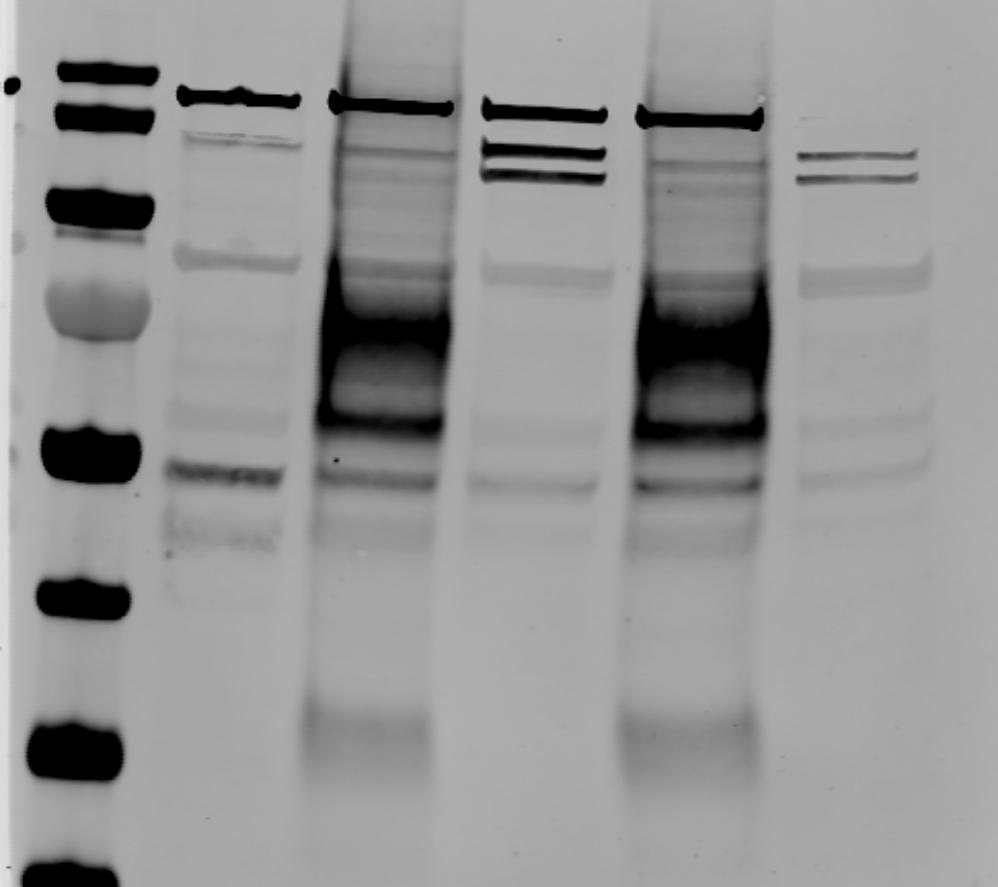


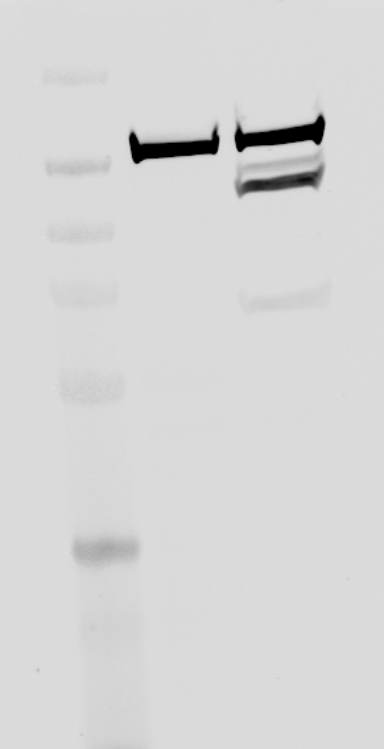

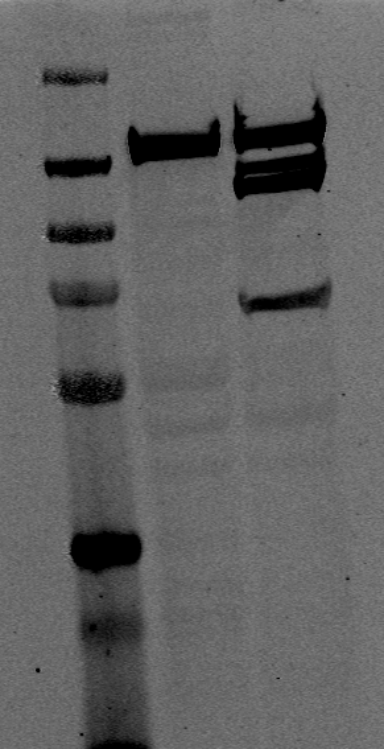

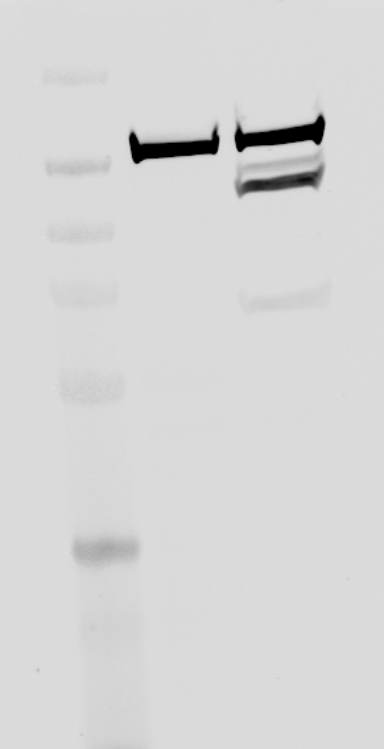

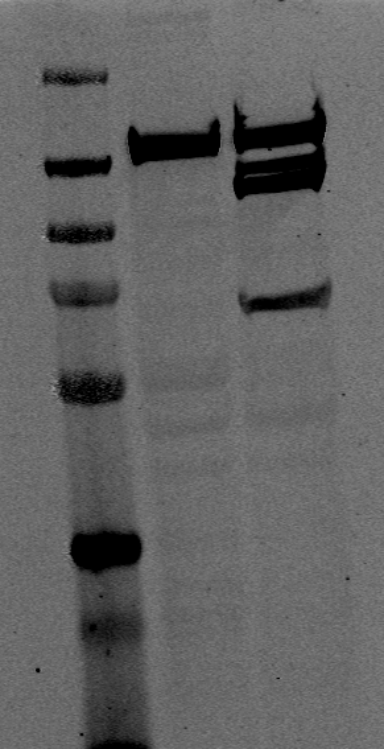


Lower exposure – 800 channel

**Figure 1- figure supplement 1F**

WT 143B vs gpASNase1 (FLAG) expressing 143B

FLAG

Vinculin

Vinculin

50 kDa

115 kDa

Raw Images
