# Supplementary material for: Mitochondrial redox adaptations enable alternative aspartate synthesis in SDH-deficient cells
Source: eLife. 2023 Mar 8;12:e78654. doi: 10.7554/eLife.78654 (PMC10027318; doi:10.7554/eLife.78654)
Supplement: Figure 2—source data 2. [file elife-78654-fig2-data2.zip › Figure 2-source data 2.docx]

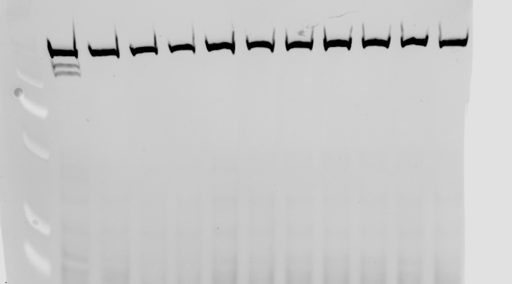

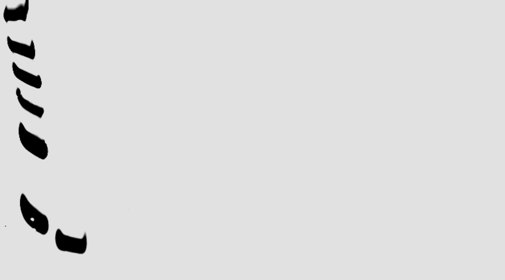

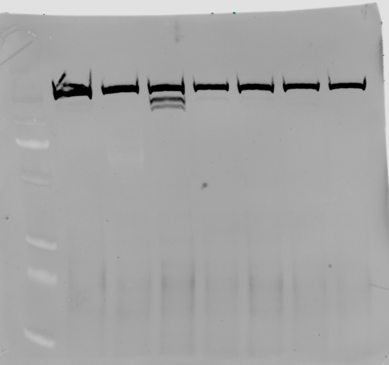

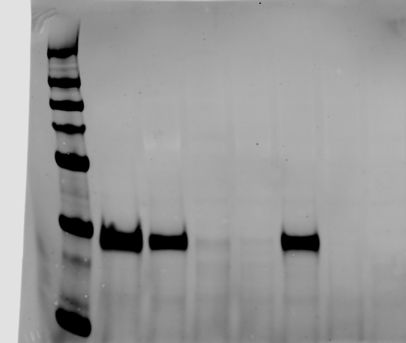


SDHB

WT SDHB KO 2

30 kDa

**Figure 2E**

WT 143B and SDHB KO clone 2 (143B)

Mock sgSDHB

**Figure 2C**

WT 143B and sgSDHB KO pool 143B

SDHB

115 kDa

Vinculin

Figure 2

30 kDa

115 kDa

Vinculin
