# Supplementary material for: Mitochondrial redox adaptations enable alternative aspartate synthesis in SDH-deficient cells
Source: eLife. 2023 Mar 8;12:e78654. doi: 10.7554/eLife.78654 (PMC10027318; doi:10.7554/eLife.78654)
Supplement: Figure 3—source data 2. [file elife-78654-fig3-data2.zip › Figure 3-source data 2.docx]

NSB = non-specific band

Figure 3

**3C**

CytoLbNOX and mitoLbNOX fractionation (FLAG, SDHB, Tubulin)

Raw Image


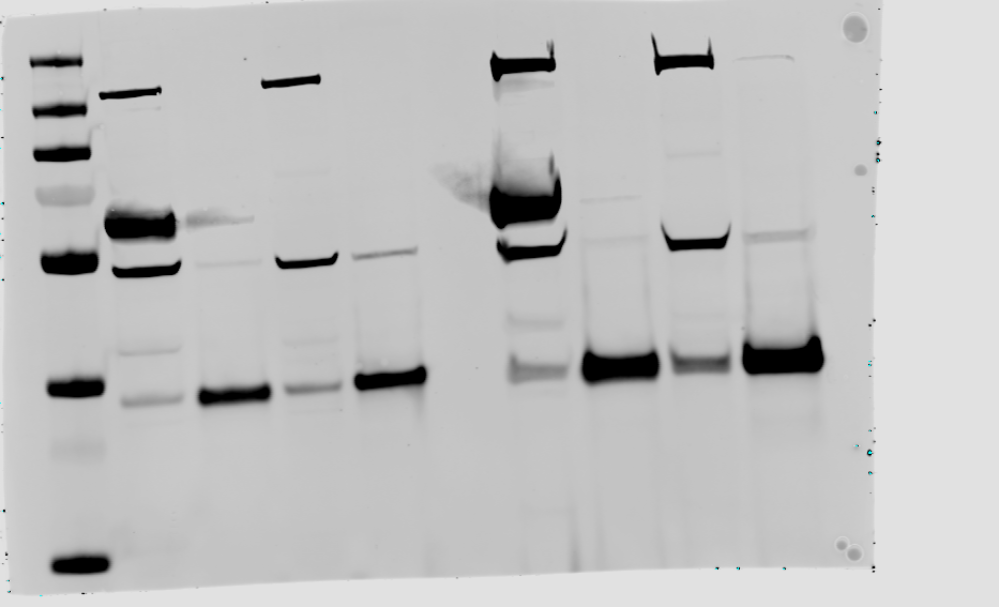


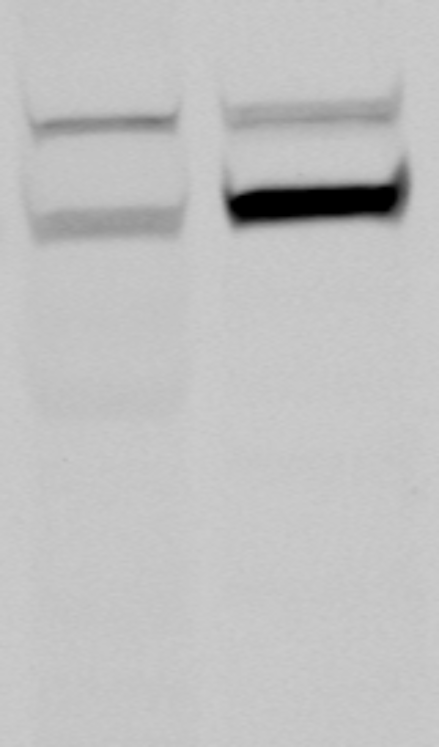

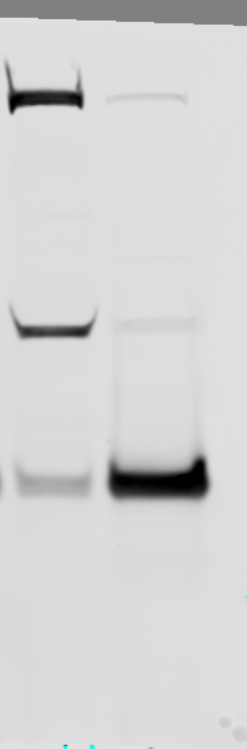

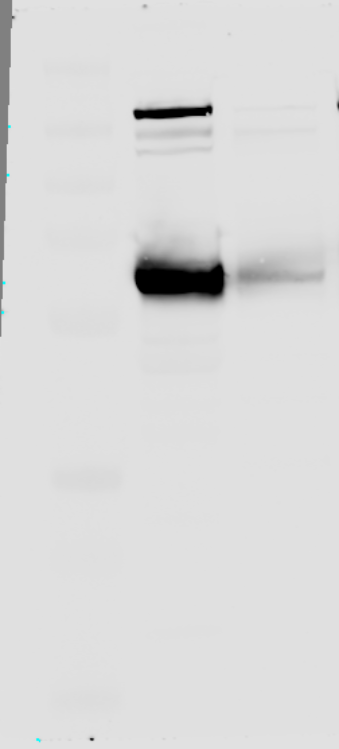

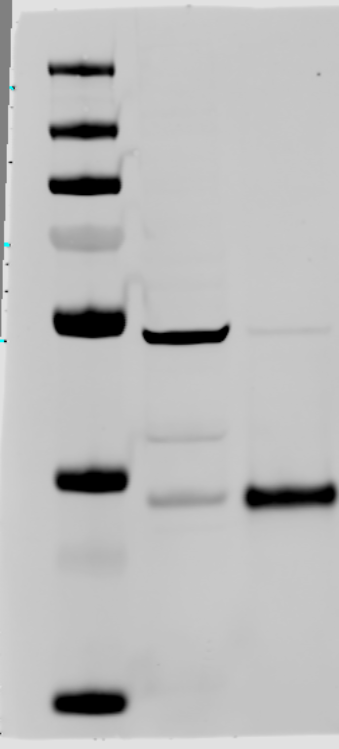


Mito fraction

Mito fraction

Cyto fraction

Cyto fraction

WT mitoLbNOX

WT mitoLbNOX

WT cytoLbNOX

WT cytoLbNOX

Vinculin

FLAG

NSB

Vinculin

SDHB

FLAG

SDHB

NSB

NSB

50 kDa

50 kDa

30 kDa

115 kDa

115 kDa

50 kDa

30 kDa

115 kDa
