# Supplementary material for: Mitochondrial redox adaptations enable alternative aspartate synthesis in SDH-deficient cells
Source: eLife. 2023 Mar 8;12:e78654. doi: 10.7554/eLife.78654 (PMC10027318; doi:10.7554/eLife.78654)
Supplement: Figure 6—source data 2. [file elife-78654-fig6-data2.zip › Figure 6-source data 2.docx]

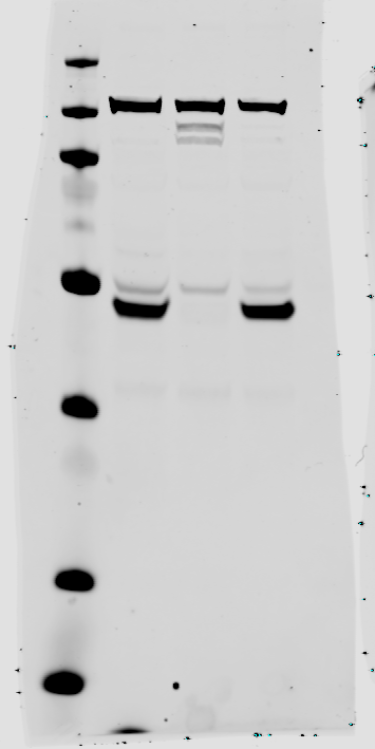


**Figure 6A**

GOT2 KO 143B blot

WT GOT2 KO GOT2 AB

Vinculin

115 kDa

50 kDa

GOT2

Raw Image

**Figure 6E**

GOT1 KO 143B blot


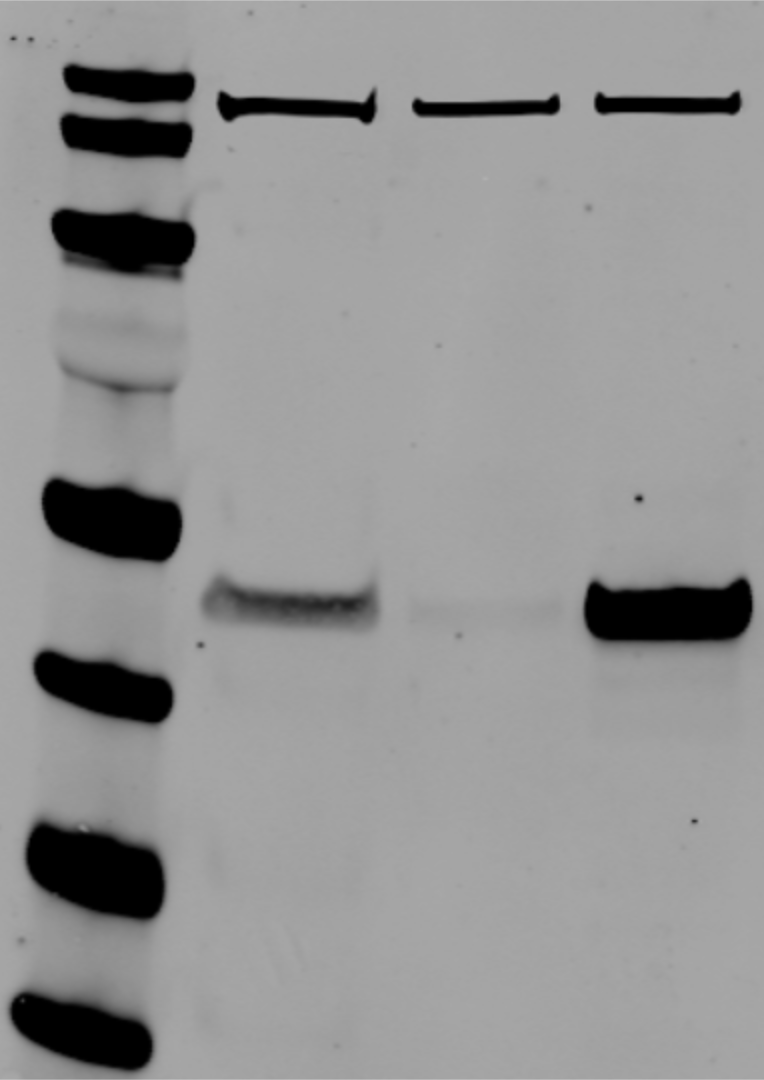

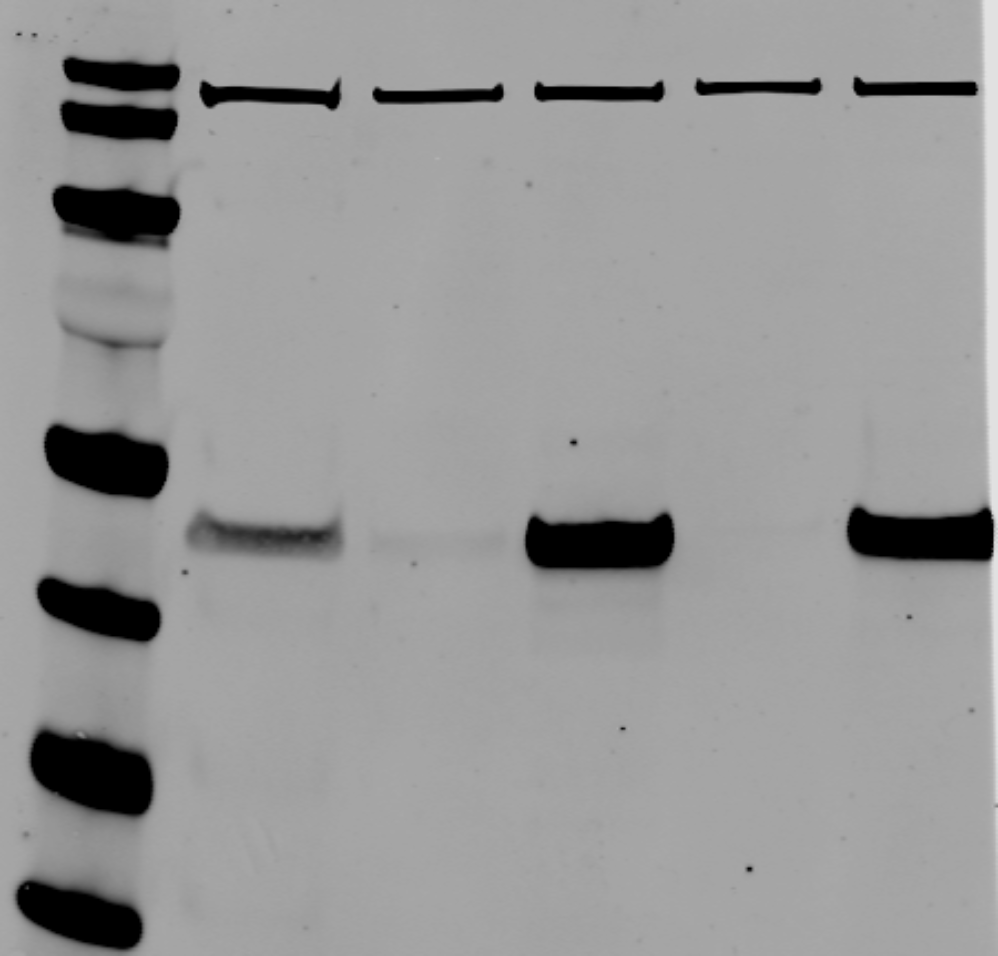


Raw Image

Vinculin

GOT1

WT GOT1 KO GOT1 AB

115 kDa

50 kDa
